# Association between cyclo-oxygenase-2 overexpression and missense *p53* mutations in gastric cancer

**DOI:** 10.1054/bjoc.2000.1607

**Published:** 2001-02

**Authors:** W K Leung, K-F To, Y-P Ng, T-L Lee, J Y W Lau, F K L Chan, E K W Ng, S C S Chung, J J Y Sung

**Affiliations:** Departments of Medicine and Therapeutics, Prince of Wales Hospital, Chinese University of Hong Kong, Shatin, Hong Kong; Departments of Anatomical and Cellular Pathology, Prince of Wales Hospital, Chinese University of Hong Kong, Shatin, Hong Kong; Departments of Surgery, Prince of Wales Hospital, Chinese University of Hong Kong, Shatin, Hong Kong

**Keywords:** cyclo-oxygenase-2, *p53* gene, mutation, gastric cancer

## Abstract

Wild-type *p53* competitively binds to the promoter region of COX-2 in vitro and inhibits its transcription. We examined the association between *p53* mutation and COX-2 expression in gastric cancer. COX-2 over-expression was seen in 19 (48.7%) cases. These tumours had more lymph-node metastasis (*P* = 0.048) and tended to have a poorer survival (*P* = 0.07). Missense mutations of *p53* were detected in 20 (51.3%) patients and had a significantly stronger COX-2 expression than tumours without *p53* mutation (*P* = 0.016). Our results suggest a link between *p53* mutation and COX-2 overexpression in gastric cancer. © 2001 Cancer Research Campaign http://www.bjcancer.com


British Journal of Cancer (2001) 84(3), 335–339
© 2001 Cancer Research Campaign
doi: 10.1054/ bjoc.2000.1607, available online at http://www.idealibrary.com on IDEAL®

### Association between cyclo-oxygenase-2 overexpression and missense *p53* mutations in gastric cancer

**WK Leung[1], K-F To[2], Y-P Ng[1], T-L Lee[2], JYW Lau[3], FKL Chan[1], EKW Ng[3], SCS Chung[3] and JJY Sung[1]**

Departments of Medicine and Therapeutics[1], Anatomical and Cellular Pathology[2], and Surgery[3], Prince of Wales Hospital, Chinese University of Hong Kong, Shatin, Hong Kong

**Summary** Wild-type *p53* competitively binds to the promoter region of COX-2 in vitro and inhibits its transcription. We examined the association between *p53* mutation and COX-2 expression in gastric cancer. COX-2 over-expression was seen in 19 (48.7%) cases. These tumours had more lymph-node metastasis ($P = 0.048$) and tended to have a poorer survival ($P = 0.07$). Missense mutations of *p53* were detected in 20 (51.3%) patients and had a significantly stronger COX-2 expression than tumours without *p53* mutation ($P = 0.016$). Our results suggest a link between *p53* mutation and COX-2 overexpression in gastric cancer. © 2001 Cancer Research Campaign http://www.bjcancer.com

**Keywords:** cyclo-oxygenase-2; *p53* gene; mutation; gastric cancer

Epidemiological studies have reported a 40–50% reduction in risk of developing colorectal cancer among chronic non-steroidal anti-inflammatory drug (NSAID) users (Thun et al, 1991; Giovannucci et al, 1994). Subsequent studies had implicated the cyclo-oxygenase-2 (COX-2) enzyme, an inducible form of prostaglandin synthase responsible for the conversion of arachidonic acid into prostaglandins, as the link between these observations (Dubois et al, 1998; Taketo, 1998). Notably, COX-2 is not just a marker of inflammation but is actively involved in the carcinogenesis process. Overexpression of COX-2 in colorectal cancer has been associated with angiogenesis (Tsujii et al, 1998), lymphatic invasion, metastasis (Tsujii et al, 1997) and poor prognosis (Sheehan et al, 1999). In this context, COX-2 overexpression is also frequently detected in gastric tumours (Ristimaki et al, 1997; Murata et al, 1999) as well as in premalignant gastric lesions (Sung et al, 2000). Similar to colonic cancer, gastric tumours with COX-2 over-expression appeared to have more frequent lymphatic invasion (Murata et al, 1999).

Nonetheless, the mechanism leading to COX-2 overexpression in tumour remains elusive. A recent in-vitro study suggested that wild-type *p53*, a major tumour suppressor gene that is involved in the control of cell cycle progression, DNA integrity and cell survival, inhibits the binding of TATA-binding proteins (TBP) to the promoter region of Cox-2 gene (Subbaramaiah et al, 1999). Thus, levels of prostaglandin $E_2$ ($PGE_2$) were 10-time lower in cells with wild-type *p53* than in those with mutated *p53*, suggesting the potential interaction of *p53* and COX-2 in cancer cells. While both *p53* mutation (Imazeki et al, 1992; Renault et al, 1993; Uchino et al, 1993) and COX-2 over-expression was frequently reported in gastric cancers, their interactions had not been properly evaluated. This study examined the correlation between *p53* mutation and COX-2 expression in gastric cancer.

*Received 7 August 2000*
*Revised 26 October 2000*
*Accepted 8 November 2000*

*Correspondence to:* WK Leung

### MATERIALS AND METHODS

### Patients

Patients with adenocarcinoma of stomach who had undergone gastrectomy in the Prince of Wales Hospital of Hong Kong were examined. A total of 39 patients were included (male:female = 21:18; median age of 69 years, ranges 29–80 years). 32 (82.1%) cases had tumours located in the distal stomach and there were 7 (17.9%) cases of proximal cancer. 22 (56.4%) of these tumours were intestinal type whilst the rest were classified as diffuse type according to Lauren classification. Early gastric cancer as defined by lesions confined to the gastric mucosa and submucosa was seen in 4 cases. All patients were regularly followed up after surgery (median 23 months, range 3–149 months). Survival was measured from the time of surgery till death.

### COX-2 immunohistochemistry

Archive pathological specimens were retrieved. 5-µm thick formalin-fixed and paraffin – embedded gastrectomy sections was retrieved. Sections were deparaffinized and endogenous peroxidase activity was blocked with 3% $H_2O_2$ in Tris-buffered saline (TBS). Non- specific binding was blocked with 5% rabbit serum (DAKO, Glostrup, Denmark) in TBS, and the tissues were incubated with antibody against COX-2 (1:100, Santa Cruz, Santa Cruz, CA) in TBS containing 2% rabbit serum and 1% bovine serum albumin. This was followed by sequential incubation with biotinylated rabbit anti-goat immunoglobulins (1:400, DAKO) and avidin–biotin peroxidase complex (DAKO) respectively. Colour was developed in DAB solution (Sigma, St Louis, MO) and counterstained with Mayer's haematoxylin. Negative control was performed by incubating samples without the primary antibody.

COX-2 expression was scored semi-quantitatively according to the percentage of positively stained tumour cells: grade 0 = no expression, 1 = <10%, 2 = 10–30%, 3 = 30–60% and 4 = >60% expression. A minimum of 10 high power view was used to assess COX-2 expression level in tumour cells (Fig. 1). Positive staining in stromal tissues and inflammatory cells was not counted. Two independent investigators (KFT and TLL) who were blinded to the *p53* mutation statuses performed the assessment. The mean expression level (in percentage) was used in subsequent analysis. In discordant cases (when inter-observer differences were greater than 30%), the two investigators would review the slides again. This immunostaining result had been validated by in-situ hybridization by using anti-sense COX-2 RNA probe (Sung et al, 2000). Over-expression of COX-2 was defined as grade 2 or above expression.

### *p53* mutation analysis

The formalin-fixed, paraffin-embedded tissues were retrieved and cut into 7 μm sections. Area containing cancer was carefully microdissected. DNA extraction was performed by using High Pure PCR Template Preparation Kit (Roche, GmbH, Germany) as described by the manufacturer. Mutations in *p53* were determined by PCR-based single strand conformational polymorphism (SSCP) of exons 5–8, where most mutations were detected. The primers used for amplification were previously reported (Hse et al, 1991; Okamoto et al, 1991). The PCR reaction mixtures contained 1 × PCR Buffer, 2.5 mM $MgCl_2$, 0.1 mM of each dNTPs, 0.25 μM of each primer, 0.625 U Taq polymerase (Gibco BRL, Rockville, MD), and 1 μl of DNA template in a 25 μl reaction volume. Nested PCR was performed and 0.3 μl (~3 μCi) of (α-$^{32}$P)dCTP (NEN, MA) was added into the second amplification. The MKN-45 human gastric cancer cell line (Riken Cell Bank, Tokyo, Japan), that has wild-type *p53* (Matozaki et al, 1992), was included as normal control.

For SSCP, 5 μl of the nested PCR products were mixed with 45 μl of loading dye (95% formamide, 0.05% xylene cyanol and 0.05% bromophenol blue). The mixture was heated to 95˚C for 10 minutes and then put in ice immediately. A 4 μl aliquot was loaded into 8% non-denaturing polyacrylamide gel with 5% glycerol. Electrophoresis of the gel was carried out at room temperature for 18 hours. The gel was dried and exposed to X-ray film at −80˚C with intensifying screen for 1 to 2 days. The presence of an abnormal band shift when compared to wild-type control was noted. Amplifications were repeated for samples showing band shifts to ensure that consistent result were obtained. The shifted bands were excised from the polyacrylamide gel and eluted by Milli-Q water. Direct DNA sequencing was performed by using ABI Prism 310 Genetic Analyzer according to standard protocol (Perkin Elmer, Branchburg, NJ). Both forward and reverse primers were used for sequencing and all positive samples were repeatedly tested.

### Statistics

All statistical calculations were performed by SPSS for Windows software (version 9.0). Fisher exact test was used for categorical data and Mann-Whitney U test was used in the comparison of COX-2 expression between patients with and without *p53* mutation. Survival data was summarized by Kaplan-Meier curve and compared by log-rank test. A *P* value (two-tailed) of less than 0.05 was considered statistically significant.

### RESULTS

### COX-2 expression

30 (74.4%) cases of gastric tumours showed COX-2 expression (grade 1 and above) while COX-2 overexpression (grade 2 and above) was detected in 19 (48.7%) cases. Tumours with COX-2 over-expression had more lymph-node metastasis (78.9% versus 45%, *P* = 0.048). However, there was no significant difference in demographic data, histological types and tumour locations between tumours with and without COX-2 over-expression (Table 1). There was a trend towards better prognosis for tumours without COX-2 overexpression (median survival 68.8 vs 28.8 months; log rank test, *P* = 0.07; Figure 2).

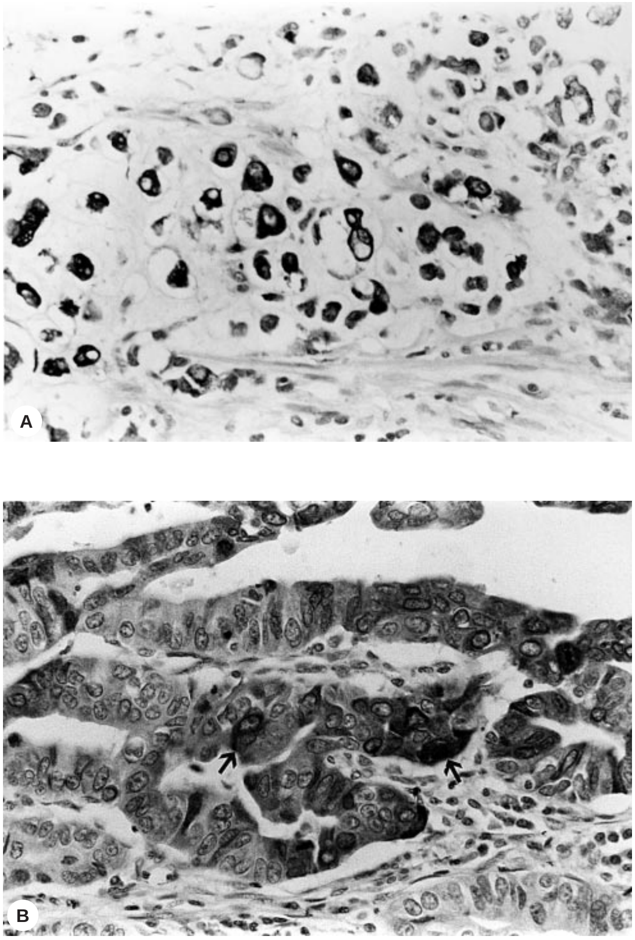

**Figure 1** (**A**) A representative sample of diffuse type gastric cancer that showed discohesive sheets of tumour cells (×400). Majority of cancer cells (>60%, grade 4) exhibited strong cytoplasmic staining for COX-2 (dark colour). (**B**) Intestinal type gastric cancer showing malignant glandular pattern. More than 10% (grade 2) of tumour cells demonstrated COX-2 expression (dark colour staining). Arrows indicated some of the positively stained cells (×400)

### *p53* mutation

*p53* mutation was detected in 20 tumours (51.3%). All mutations were missense mutations leading to amino acid substitutions (Table 2). Mutations in exon 5 were the most commonly detected (60%), followed by mutations in exon 7 (25%). 18 (90%) of these mutations were G:C → A:T transition. Gastric tumours with missense *p53* mutations had a significantly higher level of COX-2 expression than tumours with wild-type p53 (median scores: 3 versus 1, *P* = 0.016, Figure 3). There was no correlation between p53 mutation and clinicopathological features of tumours including age of patients, types of tumor, presence of lymph node metastasis and survival (Table 1).

 © 2001 Cancer Research Campaign

**Table 1** Association of COX-2 expression and p53 mutation with clinicopathological features of gastric cancer

| | COX-2 expression level | | | *p53* mutation | | |
|---|---|---|---|---|---|---|
| | 2–4 (*n* = 19) | 0–1 (*n* = 20) | *P* | Present (*n* = 20) | Absent (*n* = 19) | *P* |
| Median age | 58 | 63 | NS[a] | 58 | 63 | NS |
| Male:female | 10:9 | 11:9 | NS | 9:11 | 12:7 | NS |
| Histological type | | | | | | |
| Intestinal | 10 | 12 | NS | 11 | 11 | NS |
| Diffuse | 9 | 8 | | 9 | 8 | |
| Tumour location | | | | | | |
| Distal | 16 | 16 | NS | 15 | 17 | NS |
| Proximal | 3 | 4 | | 5 | 2 | |
| Early cancer | 1 | 3 | NS | 2 | 2 | NS |
| Lymphatic involvement | 15 | 9 | 0.048 | 15 | 9 | NS |

[a]NS = not significant.

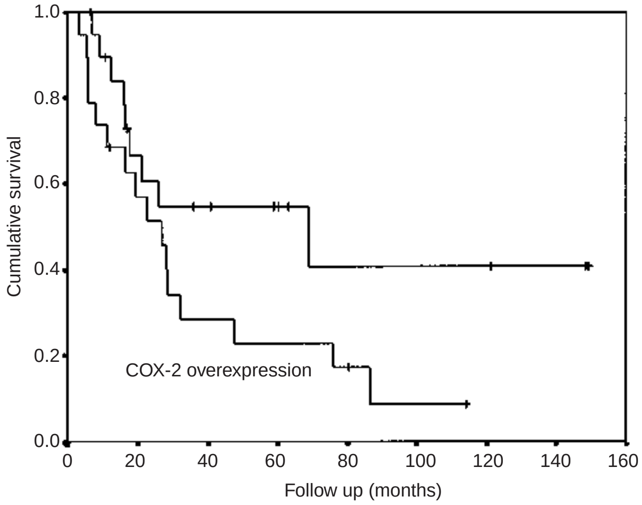

**Figure 2** Kaplan–Meier curve of gastric cancer with and without COX-2 overexpression. The upper line represented tumours without COX-2 overexpression (grade 0 or 1) whilst the lower line indicated cancers with COX-2 overexpression (median survival 28.8 months versus 68.8 months; *P* = 0.07, log rank test)

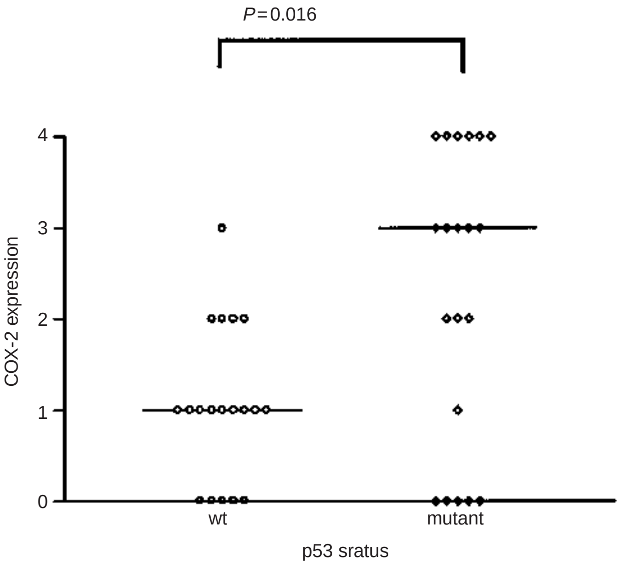

**Figure 3** The scattergram of COX-2 expression in *p53* mutated tumours (mutant) versus tumours without wild-type *p53* (wt). The horizontal line represents the median value of COX-2 expression

### DISCUSSION

The role of wild-type *p53* protein is multiple and expression of high level of *p53* results in cell cycle arrest and apoptosis. Furthermore, wild-type *p53* could serve as a transcriptional activator of genes containing the *p53*-binding sites as well as inhibiting gene transcription (Ko and Prives, 1996). Thus, mutations in the core domain of *p53* may alter its binding ability and affect its role in transcriptional regulation. One of the ways by which *p53* inhibits gene expression is via interaction with the TATA-binding proteins (TBP) and thereby interfering with the assembly of a functional transcription initiation complex (Seto et al, 1992; Mack et al, 1993). Accordingly, a recent in-vitro study demonstrated that wild-type *p53* inhibits the formation of the complex between TBP and human Cox-2 promoters in a cell-free system (Subbaramaiah et al, 1999). Intuitively, *p53* mutation would result in loss of inhibitory effect on COX-2 expression in human cancer.

We showed that tumours with *p53* missense mutation, which leads to amino acid substitution, had a higher level of COX-2 expression when compared to tumours without *p53* mutation. To our knowledge, this is the first report of this correlation in human cancer. In contrast to other tumour-related genes such as the oncogenes of the *ras* family, mutations in 'hotspot' codons accounted for only about 20% of all *p53* mutations reported so far. As in this study, mutation in codons 175 and 248 were detected in 3 cases only (14.3%) and diverse mutation patterns were observed for the remaining samples. Notably, most of these mutations were G:C → A:T transition as previously reported (Imazeki et al, 1992; Renault et al, 1993). This transition has been linked to exposure to nitrous oxide (Nguyen et al, 1992). On the other hand, with the diverse mutation patterns, it is not difficult to anticipate the heterogeneous COX-2 expression level among tumours with different *p53* mutations. It would be interesting to study the in-vitro promoter activity and responses by using reporter assays or DNA-binding assays with various forms of *p53* variants.

© 2001 Cancer Research Campaign

**Table 2** Summary of *p53* mutation in gastric cancer cases

| Case no | Site | Type | COX-2 expression | *p53* mutation | Exon | Codon | Mutation | Amino acid |
|---|---|---|---|---|---|---|---|---|
| 3 | A | Diffuse | 0 | No | | | | |
| 9 | A | Diffuse | 1 | Yes | 5 | 140 | ACC>ATC | Thr>Ile |
| 10 | A | Intestinal | 1 | No | | | | |
| 11 | A | Intestinal | 4 | Yes | 5 | 175 | CGC>CAC | Arg>His |
| 18 | B | Intestinal | 0 | No | | | | |
| 24 | B | Intestinal | 1 | No | | | | |
| 26 | A | Intestinal | 2 | Yes | 5 | 129 | GCC>GTC | Ala>Val |
| 28 | A | Intestinal | 1 | No | | | | |
| 31 | A | Intestinal | 1 | No | | | | |
| 33 | A | Intestinal | 2 | No | | | | |
| 36 | A | Intestinal | 3 | Yes | 5 | 146 | TGG>CGG | Trp>Arg |
| 44 | C | Intestinal | 0 | No | | | | |
| 47 | A | Intestinal | 2 | No | | | | |
| 50 | C | Intestinal | 0 | Yes | 5 | 154 | GGC>AGC | Gly>Ser |
| 53 | B | Diffuse | 4 | Yes | 7 | 248 | CGG>CAG | Arg>Gln |
| 55 | B | Diffuse | 4 | Yes | 5 | 181 | CGC>TGC | Arg>Cys |
| 57 | A | Diffuse | 2 | No | | | | |
| 59 | B | Diffuse | 2 | Yes | 5 | 155 | ACC>ATC | Thr>Ile |
| 62 | B | Diffuse | 4 | Yes | 7 | 250 | CCC>CTC | Pro>Leu |
| 70 | A | Intestinal | 0 | Yes | 5 | 146 | TGG>CGG | Trp>Arg |
| 72 | C | Diffuse | 4 | Yes | 5 | 175 | CGC>CAC | Arg>His |
| 77 | C | Diffuse | 1 | No | | | | |
| 78 | A | Diffuse | 0 | Yes | 5 | 170 | ACG>ATG | Thr>Met |
| 80 | C | Intestinal | 0 | Yes | 8 | 262 | GGT>AGT | Gly>Ser |
| 90 | C | Intestinal | 3 | Yes | 5 | 171 | GAG>AAG | Glu>Lys |
| 93 | A | Intestinal | 3 | Yes | 6 | 192 | CAG>CAC | Gln>His |
| 99 | A | Diffuse | 3 | No | | | | |
| 101 | B | Diffuse | 0 | Yes | 8 | 267 | CGG>CAG | Arg>Gln |
| 102 | B | Intestinal | 1 | No | | | | |
| 104 | B | Intestinal | 1 | No | | | | |
| 106 | A | Diffuse | 4 | Yes | 5 | 177 | CCC>CTC | Pro>Leu |
| 108 | A | Intestinal | 3 | Yes | 6 | 193 | CAT>AAT | His>Asn |
| 110 | A | Diffuse | 0 | No | | | | |
| 112 | C | Intestinal | 2 | Yes | 7 | 230 | ACC>GCC | Thr>Ala |
| 115 | A | Diffuse | 1 | No | | | | |
| 116 | A | Intestinal | 3 | Yes | 7 | 245 | GGC>AGC | Gly>Ser |
| 117 | A | Intestinal | 0 | No | | | | |
| 120 | A | Diffuse | 1 | No | | | | |
| 121 | A | Diffuse | 2 | No | | | | |

A = antrum, B = body, C = cardia.

In this study, COX-2 expression was also detected in gastric tumour with wild-type *p53*. This finding is not unexpected since other factors are also involved in regulation of COX-2 expression. For example, *H. pylori*-associated gastritis has been associated with upregulation of COX-2 (Fu, 1999). This is turn may be triggered by pro-inflammatory factors such as cytokines, tumour necrosis factor-α, and nuclear factor-κB (Dubois et al, 1998). Recently, the *APC* gene was also found to play a role in the translational regulation of COX-2 in colorectal cancer cell line (Hsi, 1999). Given the complexity of regulation of COX-2 expression, wild-type *p53* is probably just one of the many factors responsible for the inhibition of COX-2 transcription in normal tissues.

A modest increase in lymphatic involvement was detected among tumours with COX-2 overexpression. In this regard, it is not surprising to observe a trend towards lower survival in these tumours when compared to tumours without COX-2 overexpression. Similar findings had been reported in colorectal as well as in gastric cancer (Murata et al, 1999; Sheehan et al, 1999). Taken together, these results suggested the prognostic significance of COX-2 in gastrointestinal tumours and thus, COX-2 is probably playing an active role in tumorigenesis and is not just an epiphenomenon.

In conclusion, we have demonstrated that gastric tumours with missense p53 mutations were associated with higher level of COX-2 expression, suggesting the potential role of wild-type *p53* in the regulation of COX-2 expression. Studies that look into the regulation of COX-2 expression in cancer may offer a new insight into gastric carcinogenesis and plausibly, chemoprevention pathways.

### REFERENCES

Dubois RN, Abramson SB, Crofford L, Gupta RA, Simon LS, van de Putte LB and Lipsky PE (1998) Cyclooxygenase in biology and disease. *FASEB J* **12**: 1063–1073

Fu S, Ramanujam KS, Wong A, Fantry GT, Drachenberg CB, James SP, Meltzer SK and Wilson KT (1999) Increased expression and cellular localization of inducible nitric oxide synthase and cyclooxygenase 2 in *Helicobacter pylori* gastritis. *Gastroenterol* **116**: 1319–1329

Giovannucci E, Rimm EB, Stampfer MJ, Colditz GA, Ascherio A and Willett WC (1994) Aspirin use and the risk for colorectal cancer and adenoma in male health professional. *Ann Int Med* **121**: 241–246

Hse IC, Metcalf RA, Sun T, Welsh JA, Wang NJ and Harris CC (1991) Mutational hotspot in the p53 gene in human hepatocellular carcinoma. *Nature* **350**: 427–428

 *© 2001 Cancer Research Campaign*

Hsi LC, Angerman-Sterwart J and Eling TE (1999) Introduction of full-length APC modulates cyclooxygenase-2 expression in HT-29 human colorectal carcinoma cells at the translational level. *Carcinogenesis* **20**: 2045–2049

Imazeki F, Omata M, Nose H, Ohto M and Isono K (1992) p53 gene mutations in gastric and esophageal cancers. *Gastroenterol* **103**: 892–896

Ko LJ and Prives C (1996) p53: puzzle and paradigm. *Gene & Development* **10**: 1054–1072

Mack DH, Vartikar J, Pipas JM and Laimins LA (1993) Specific repression of TATA-mediated but not initiator-mediated transcription by wild-type p53. *Nature* **363**: 281–283

Matozaki T, Sakamoto C, Matsuda K, Suzuki T, Konda Y, Nakano O, Wada K, Uchida T, Nishisaki H, Nagao M and Kasuga M (1992) Missense mutations and a deletion of the p53 gene in human gastric cancer. *Biochem Biophy Res Comm* **182**: 215–223

Murata H, Kawano S, Tsuji S, Tsujii M, Sawaoka H, Kimura Y, Shiozaki H and Hori M (1999) Cyclooxygenase-2 over-expression enhances lymphatic invasion and metastasis in human gastric carcinoma. *Am J Gastroenterol* **94**: 451–455

Nguyen T, Brunson D, Crespi CL, Penman BW, Wishnok JS and Tannenaum SR (1992) DNA damage and mutation in human cells exposed to nitric oxide in vitro. *Proc Natl Acad Sci USA* **89**: 3030–3034

Okamoto A, Sameshima Y, Yokoyama S, Terashima Y, Sugimura T, Terada M and Yokota J (1991) Frequent allelic losses and mutations of the p53 gene in human ovarian cancer. *Cancer Res* **51**: 5171–5176

Renault B, van den Broek M, Fodde R, Wijinen J, Pellegata NS, Amadori D, Khan PM and Ranzai GN (1993) Base transitions are the most frequent genetic changes at p53 in gastric cancer. *Cancer Res* **53**: 2614–2617

Ristimaki A, Honkanen N, Jankala H, Sipponen P and Harkonen M (1997) Expression of cyclooxygenase-2 in human gastric carcinoma. *Cancer Res* **57**: 1276–1280

Seto E, Usheva A, Zambetti GP, Momand J, Horikoshi N, Weinmann R, Levine AJ and Shenk T (1992) Wild-type p53 binds to the TATA-binding protein and represses transcription. *Proc Natl Acad Sci USA* **89**: 12028–12032

Sheehan KM, Sheehan K, O'Donoghue DP, MacSweeney F, Conroy RM, Fitzgerald DJ and Murray FE (1999) The relationship between cyclooxygenase-2 expression and colorectal cancer. *JAMA* **282**: 1254–1257

Subbaramaiah K, Altorki N, Chung WJ, Mestre JR, Sampat A and Dannenberg AJ (1999) Inhibition of cyclooxygenase-2 gene expression by p53. *J Biol Chem* **274**: 10911–10915

Sung JJY, Leung WK, Go MYY, To KF, Cheng ASL, Ng EKW and Chan FKL (2000) COX-2 expression in *H. pylori*-associated premalignant and malignant gastric lesions. *Am J Pathol* 2000; **157**: 729–735

Taketo MM (1998) Cyclooxygenase-2 inhibitors in tumorigenesis (Part II). *J Natl Cancer Inst* **90**: 1609–1620

Thun MJ, Namboodiri MM and Health CW Jr (1991) Aspirin use and reduced risk of fatal colon cancer. *N Engl J Med* **325**: 1593–1596

Tsujii M, Kawano S and DuBois RN (1997) Cyclooxygenase-2 expression in human colon cancer cells increases metastatic potential. *Proc Natl Acad Sci USA* **94**: 3336–3340

Tsujii M, Kawano S, Tsuji S, Sawaoka H, Hori M and DuBois RN (1998) Cyclooxygenase regulates angiogenesis induced by colon cancer cells. *Cell* **93**: 705–716

Uchino S, Noguchi M, Ochiai A, Saito T, Kobayashi M and Hirohashi S (1993) p53 mutation in gastric cancer: a genetic model for carcinogenesis is common to gastric and colorectal cancer. *Int J Cancer* **54**: 759–764

*© 2001 Cancer Research Campaign*
